# Supplementary material for: Antimicrobial efficacy and biocompatibility of extracts from Cryptocarya species
Source: PLoS One. 2021 Dec 31;16(12):e0261884. doi: 10.1371/journal.pone.0261884 (PMC8719776; doi:10.1371/journal.pone.0261884)
Supplement: Attachment — Submitted filename: Response to Reviewers.doc [file pone.0261884.s001.doc]

### Dr. Janaina Habib Jorge

Department of Dental Materials and Prosthodontics

##### Araraquara Dental School – UNESP

Rua Humaitá n^0^ 1680

ARARAQUARA - SÃO PAULO - BRAZIL

c.e.p. - 14801-903

Phone: #55#016# 33016550

e-mail adresses: habib.jorge@unesp.br

November 2^th^ 2021

**Editor-in-Chief**

**Dear Professor**

Thank you for your considerations on the manuscript **“*Antimicrobial efficacy and biocompatibility of extracts from Cryptocarya species*”**. We are submitting the revised manuscript with the changes highlighted with “Track Changes”. We would like to affirm our great interest in publishing the article in this respected journal and we believe that this new version of the manuscript is now suitable for publication.

**Journal Requirements:**

Please remove any funding-related text from the manuscript and let us know how you would

like to update your Funding Statement.

**Response:** The funding-related text from manuscript was removed and we confirm that *“The funders had no role in study design, data collection and analysis, decision to publish, or preparation of the manuscript.”*

**Comments to the Author**

**Reviewer #1:** The paper describes the fungicidal effect of *Cryptocarya* spp extracts on planktonic form and biofilm grown onto dental resin of *Candida albicans*. The paper also describes the cytocompatibility of the extract on oral keratinocytes. The Authors tested *C. moschata, C. saligna* and *C. mandioccana* solutions at various concentrations in comparison with nystatin solution at 100,000 IU /mL and phosphate buffered saline (PBS) solution. The results showed the ability of the extract to inhibit the fungal growth but at the same time they revealed its cytotoxicity. *C. saligna* had no effect on *C. albicans*. The paper is clear, and the experiments correctly done.

**Firstly, we would like to thank you for your considerations on the manuscript. All questions and considerations have been answered, as follows:**

- The paper is not fully informative, and the composition of the extract should be determined to understand the differences among the different *Cryptocarya* species and sources (leaves, nuts etc).

**Response:** The analysis of plant extracts by UPLC-DAD-MS was performed and details about the methodology were added as suggested.

- Figures should be included indication of statistical significance where relevant.

**Response:** The indication of statistical significance as included as suggested.

**Reviewer #2:**

**Firstly, we would like to thank you for your considerations on the manuscript. All questions and considerations have been answered, as follows:**

- spp is not written in italics and as it is an abbreviation.

**Response:** The abbreviation was corrected in all the text as suggested.

- The presentation of the extract preparation is poorly described. Was the plant material dried? If so, how, where, for how long and at what temperature? What is the concentration of the hydroalcoholic mixture for the extraction? To remove the solvent by lyophilization, first the ethanol must be removed with the aid of a rot evaporator and then the residual water can be frozen and lyophilized. Was it possible to freeze the hydroalcoholic extract obtained to be later lyophilized?

**Response:** Details about the methodology were added as suggested.

- In the statement "The crude extracts were weighed and aliquots containing adequate but were then diluted in water with ethanol in the proportions of interest", can a hydroalcoholic mixture be used to verify the antimicrobial action? Wouldn't the presence of alcohol be toxic? Therefore, action is to be expected in rehearsals and hence it is not valid.

**Response:** For microbiological analyses, the crude extracts were weighed and aliquots and, then, diluted in phosphate bufferid saline (PBS) with ethanol (5%) in the proportions of interest. This concentration is not toxic to oral cells*.

**Calderón-Montaño JM, Jiménez-Alonso JJ, Guillén-Mancina E, Burgos-Morón E, López-Lázaro M. A 30-s exposure to ethanol 20% is cytotoxic to human keratinocytes: possible mechanistic link between alcohol-containing mouthwashes and oral cancer. Clin Oral Investig. 2018 Nov;22(8):2943-2946.*

- For the preparation of *Cryptocarya* species concentrations, what does the *Cryptocarya* extracts 100% mean? Was undiluted extract used?

**Response:** The extract 100% is the undiluted extract.

Best regards.

Sincerely,

Janaina Habib Jorge
